# Near-peers improve patient safety training in the preclinical curriculum

**DOI:** 10.1080/10872981.2017.1289315

**Published:** 2017-02-21

**Authors:** Sally R. Raty, Cayla R. Teal, Elizabeth A. Nelson, Anne C. Gill

**Affiliations:** ^a^ Department of Anesthesiology and Perioperative Medicine, The University of Texas MD Anderson Cancer Center, Houston, TX, USA; ^b^ Department of Medicine, Assistant Dean for Academic Affairs, Texas A&M Health Science Center, Round Rock, TX, USA; ^c^ Senior Associate Dean for Undergraduate Medical Education, The University of Texas at Austin, Dell Medical School, Austin, TX, USA; ^d^ Departments of Pediatrics and Medical Ethics, Baylor College of Medicine, Houston, TX, USA

**Keywords:** Residents as teachers, educational continuum, curricular innovation, curriculum development, undergraduate medical education

## Abstract

**Background**: Accrediting bodies require medical schools to teach patient safety and residents to develop teaching skills in patient safety. We created a patient safety course in the preclinical curriculum and used continuous quality improvement to make changes over time.

**Objective**: To assess the impact of resident teaching on student perceptions of a Patient Safety course.

**Design**: Using the Institute for Healthcare Improvement patient safety curriculum as a frame, the course included the seven IHI modules, large group lectures and small group facilitated discussions. Applying a social action methodology, we evaluated the course for four years (Y1–Y4).

**Results**: In Y1, Y2, Y3 and Y4, we distributed a course evaluation to each student (*n* = 184, 189, 191, and 184, respectively) and the response rate was 96, 97, 95 and 100%, respectively. Overall course quality, clarity of course goals and value of small group discussions increased in Y2 after the introduction of residents as small group facilitators. The value of residents and the overall value of the course increased in Y3 after we provided residents with small group facilitation training.

**Conclusions**: Preclinical students value the interaction with residents and may perceive the overall value of a course to be improved based on near-peer involvement. Residents gain valuable experience in small group facilitation and leadership.

## Introduction

The Association of American Medical Colleges (AAMC) and the Lucian Leape Institute recommend incorporating patient-safety education into medical school curricula.[1,2] However, creating a course on the nascent science of patient safety is fraught with challenges, including having a sufficient number of faculty with adequate expertise in the discipline. Since 2002, residents have been trained within the framework of the Accreditation Council for Graduate Medical Education (ACGME) core competencies and should have both an understanding of a systems-based approach to care delivery and an awareness of patient-safety issues.[3] In addition, the ACGME and the Liaison Committee on Medical Education (LCME) mandate that residents be provided opportunities to effectively educate students and other healthcare professionals.[4,5] A recent review of the literature on residents as teachers of near-peers and as role models [6–8] indicates a need for rigorously evaluated, educational training interventions that are reproducible.

We designed a patient safety curriculum (PSC) for second-year medical students (MS2 s) based on the seven online patient safety modules from the Institute for Healthcare Improvement (IHI):[9] Introduction to Patient Safety; Fundamentals of Patient Safety; Human Factors and Safety; Teamwork and Communication; Culture of Safety; Responding to Adverse Events; and Root Cause Analysis. In addition, the PSC includes a large-group orientation session, a lecture on the fundamentals and culture of patient safety, and five resident-facilitated small groups (8–10 MS2s) with case-based discussions.

We describe herein the evolution of our PSC over the course of four years, examining how changes in small group facilitation from student peers to residents changes student perceptions of the PSC and describing how to successfully include residents as teachers in the preclinical curriculum. We postulate that residents as near-peers would be ideal small-group facilitators able to contextualize patient-safety concepts for preclinical students.

## Methods

We requested IRB approval for this effort and received an exemption because our project was deemed to be ‘routine program evaluation.’

### Course development

In Y1, we recruited eight MS2 peer-teaching assistants (PTAs) from the Baylor College of Medicine (BCM) Patient Safety Interest Group to assist with the small-group discussion portion of each PSC class. PTAs completed all seven IHI Patient Safety modules before the start of the PSC, circulated among 30 discussion groups, answered questions, and provided content information. PTAs were not assigned to specific groups as facilitators or teachers. PTAs met with the Course Director after each class and at the conclusion of the PSC for debriefing. Based on evaluation data from MS2s in Y1, we modified the teaching strategies for Y2.

In Y2, the PTAs were replaced with 18 resident teaching assistants (RTAs). Otherwise, the course was unchanged. The Course Director recruited RTAs via an email sent to all Program Directors. Residents and fellows above the PGY-1 level could self-nominate or be nominated by a Program Director. Nominated RTAs were accepted into the program if their Program Directors signed an agreement providing assurance that the respective resident(s) would be released from clinical activities to participate in the PSC. They also completed the IHI Patient Safety modules, participated in a one-hour course overview, and attended the seven PSC sessions. During the course overview, RTAs received an introductory lecture on patient safety, learned their PSC responsibilities, and received general teaching tips on small-group facilitation. Each RTA served as a discussion facilitator and content resource to two groups of six MS2s. RTAs evaluated student participation as a component of the course grade, and students evaluated their specific RTA’s performance and effectiveness.

We emailed reminders to the RTAs and monitored their module completion. RTAs debriefed with the Course Director at the midway point and completion of the course. At the end of the course, MS2s participated in a large-group, inter-professional session and submit evaluations of the course and their small group leaders using E*Value,[10] a curriculum management software program. In Y2, MS2s used a seven-point rating scale, with one equal to *strongly disagree* and seven equal to *strongly agree*, to evaluate the course and its facilitators.

In Y3, we modified the course teaching strategy, based on evaluations from MS2s and feedback from RTAs. We recruited 27 RTAs, including five residents who served as RTAs in Y2 (the other 13 RTAs from Y2 had graduated). In Y3, each RTA facilitated a single group of 8–10 MS2s. We eliminated five large-group lectures (leaving two) and modified the clinical scenarios, effectively making the residents the central teachers for the course. As before, RTAs were required to complete the seven IHI Patient Safety modules and, in addition, attend a 1.5-hour workshop on small-group facilitation provided by a ‘master teacher’ physician. The training included strategies for effective facilitation, such as seating arrangements, structured activities, and the Tuckman Model [11] of small group development. These strategies were modeled by the master teacher during the session, and the residents practiced these techniques during role play. At the end of the Y3 course, RTAs and MS2s again completed evaluations.

In Y4, we recruited 25 RTAs. Each RTA was assigned a single group of MS2s and required to meet the same training expectations as we had in Y3. We added one inter-professional, large-group session that included nursing and pharmacy students but made no other changes to the course.  Table 1 illustrates changes in the course design from Y1-Y2.

### Course evaluations

In Y1–Y4, students rated four course attributes: (a) IHI module effectiveness; (b) clarity of learning objectives; (c) impact of small group discussions; and (d) overall course quality. In Y2–4, two outcomes and one course measure were added to the course evaluation: the impact of the course on their awareness of patient safety issues, self-confidence in reporting a patient safety concern to appropriate personnel, and overall effectiveness of the facilitators, respectively. In Y2–Y4, MS2s were asked to consider a variety of individual residents’ behaviors (e.g. preparation for the group, respect, encouragement of learners, provision of feedback) when assessing overall RTA effectiveness as a small group leader.

We used parametric procedures (analysis of variance) or the non-parametric counterparts to examine differences in each year’s cohorts’ ratings of these quantitative items and used SPSS [12] for analyses of quantitative data. Responses from pre- to post-test were analyzed using the Wilcoxon signed-rank test. Effect sizes were calculated using eta squared.

## Results

**Figure 1.**
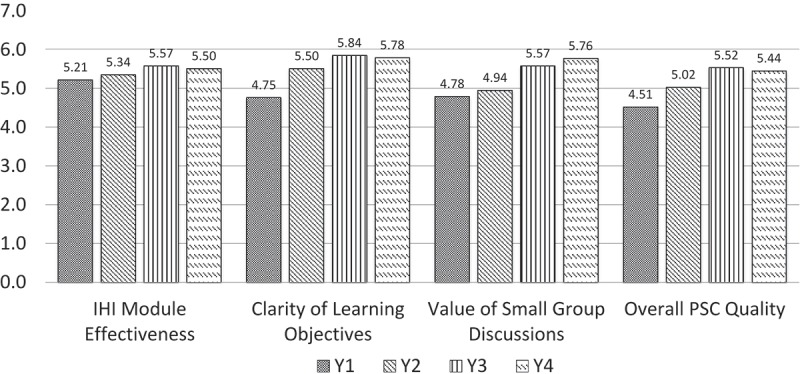
Course ratings by year.

(Figure 1). As seen in Figure 1, the ratings of the effectiveness of the IHI modules remained stable over the four years. From Y1 to Y2, when the primary change in the PSC was the replacement of PTAs with RTAs, ratings of the clarity of learning objectives and overall quality of the PSC significantly improved, but the value of small-group discussions did not improve. From Y2 to Y3, evaluations of the value of the small-group discussions, the overall course quality, and the overall effectiveness of the RTAs significantly improved for eight of the 10 individual ratings (Table 2).

**Table 1.** Patient safety course structure by year.

Demographics	Year 1 (2011)	Year 2 (2012)	Year 3 (2013)	Year 4 (2014)
Total number of students	184	189	191	184
Number of student responses	177	184	181	184
Student response rate	96%	97%	95%	100%
Teaching assistants (TA)	Student Peer	Resident	Resident	Resident
Number of TAs	8	18*	27*	25*
Number of small groups	30	32	19	20
Number of students per group	6	12	8–10	8–10
**Teaching modalities**	Modified team learning	Modified team learning	2 didactic, 5 PBL small group	2 didactic, 5 PBL small group, IPE session
	IHI modules	IHI modules	IHI modules	IHI modules
**Training**	Orientation, weekly and end of course debriefing	Orientation, small group facilitation teaching tips, weekly and end-of-course debriefing	Orientation, formal small group facilitation training, weekly email updates, mid-and end-of-course debriefing.	Orientation, formal small group facilitation training, weekly email updates, mid-and end-of-course debriefing.

**Table 2.** Comparison of ratings of small group facilitators (RTAs) by academic year.

My small group facilitator (RTA):	AY 12–13	AY 13–14	AY 14–15	Sig*	Effect size (η^2^)
	(*N* = 186)	(*N* = 188–191)	(*N* = 187–191)		
Was prepared for sessions	6.57	6.80	6.69	0.004	0.021
Encouraged learners to justify thought processes.	6.48	6.70	6.63	0.025	0.014
Created an atmosphere in which learners felt comfortable.	6.70	6.79	6.70	ns	
Was respectful of learners.	6.73	6.89	6.78	0.006	0.014
Provided feedback that helped me improve.	6.34	6.55	6.46	ns	
Kept the group focused on achieving objectives.	6.47	6.75	6.60	0.001	0.020
Gave the right amount of structure and guidance to the group.	6.38	6.72	6.67	0.003	0.028
Encouraged learners to examine different aspects of a case.	6.44	6.71	6.71	0.005	0.025
Helped to identify problems in cases and encouraged dealing with them as appropriate.	6.52	6.75	6.70	0.011	0.019
Enhanced my understanding of the clinical application of patient safety concepts.	5.76	6.75	6.72	< 0.001	0.160
Overall, the facilitator was an effective small group leader.	6.51	6.78	6.66	0.013	0.016

*Analysis of variance, Welch test

**Figure 2.**
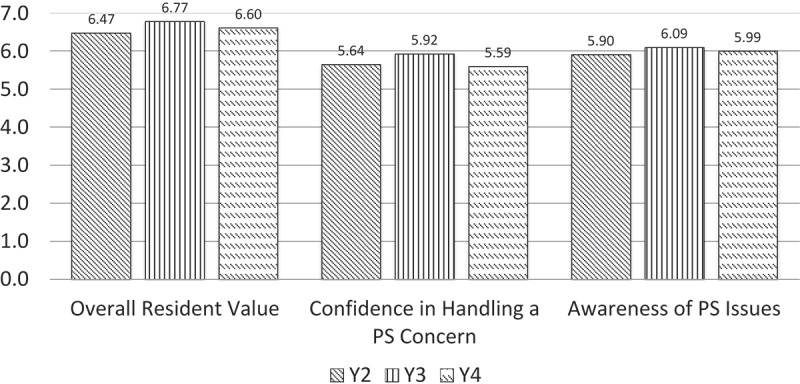
Resident ratings by year.

We also expected that as Y4’s training was not substantially different from that of Y3’s, any significant changes from year to year would be as result only of Y2 to Y3 differences, with no significant differences between Y3 and Y4.

## Discussion

Since 2011, we have required MS2s to successfully complete 10.5 h of patient-safety training. We modified the PSC each year based on data gathered from learners. Formatting the PSC around small-group discussions requires a large number of facilitators; however, initial efforts to recruit faculty yielded only five volunteers. Productivity demands on clinical faculty create formidable recruiting challenges, especially for a PSC because many current faculty were trained before patient safety became a science and may lack expertise in the subject. By engaging RTAs in the PSC, we partnered with our GME colleagues to fulfill a requirement for residents to develop teaching skills, participate in education, and work in inter-professional teams to enhance patient safety.[4]

From Y2 to Y4, 48 residents participated in at least one small-group session. These residents included trainees in post-graduate years one through eight, representing internal medicine, pediatrics, psychiatry, anesthesiology, emergency medicine, general surgery, and several surgical subspecialties, as well as a number of trainees in advanced fellowships. During the annual post-course debriefings, many residents indicated that their participation in the PSC was based on a desire to teach medical students outside the clinical setting, and almost all would recommend participation in the PSC to fellow residents.

When we started this project, we hypothesized that the changes in small group leadership from student peers to resident near-peers would improve the PSC. We also hypothesized that RTAs would increase their comfort with patient safety topics, enhance their confidence as teachers, and improve their confidence as role models for students and peers. To test this hypothesis, we compared MS2 responses in Y1 through Y4 on the course and resident evaluations. We expected the MS2 ratings to improve with the change from using PTAs to RTAs (from Y1 to Y2), and with small-group facilitation training (from Y2 to Y3). We also expected that the students’ ratings of a resident’s overall effectiveness as a facilitator would improve with increased RTA training in small-group facilitation. Our secondary analyses included assessing the impact of course changes on student awareness of patient safety issues and on their confidence in handling patient safety issues.

When we introduced the RTAs into the preclinical setting in Y2, MS2s rated the PSC significantly higher than did students from the course’s inaugural year, when PTAs were used. The improved ratings of RTAs as facilitators in Y3 compared to Y2 can be attributed to the additional training in small-group facilitation and smaller group size, rather than to individual resident’s improvement, because 72% of the Y3 RTAs did not participate in Y2 (see Figure 2). We are not able to conclude that residents are preferred to faculty members when teaching patient safety because we did not engage the faculty in the small-group section of the PSC. However, residents’ ratings meet or exceed faculty ratings compared to other preclinical courses that also use a small-group format. Our results could be limited by the possibility of differences among the four medical school cohorts involved in the PSC. Therefore, we analyzed the cohort ratings of other stable courses and found no differences in judgments about the PSC when compared to other courses.

Although costs are minimal, incorporating RTAs into the preclinical curriculum poses a unique set of challenges. Managing RTAs adds approximately two hours per week to the Course Director’s efforts during recruitment and training and another one hour per week in communication and monitoring IHI module completion for the remainder of the course. Clinical demands on residents can be considerable, and in Y4 some RTAs struggled to attend the small-group discussions. By training a pool of substitute facilitators, we minimized this impact on students. Lack of consistent attendance by some residents may explain the slight decline in the Y4 ratings of both the value of residents and the PSC, although this decline was not significant. RTAs are with the institution for a limited time, and upon their graduation, we lose an experienced PSC facilitator. While we lament the loss of exceptional RTAs, annual training is a fact of life for any course utilizing a large number of small-group facilitators and does not pose an undue burden. When we weigh the pros and cons of utilizing RTA facilitators, we posit that augmenting residents’ knowledge of patient safety science, improving residents’ small-group facilitation skills, and increasing students’ satisfaction associated with the course are value added. Using resident near-peers as small-group facilitators is an effective method for introducing patient safety concepts to medical students and for enhancing resident teaching skills. Although our study focused on a required pre-clinical PSC, this format may be applicable to other pre-clinical subjects. Future research on the resident’s perceptions of the value-added from serving as a small group facilitator may have implications for resident education.

## References

[CIT0001] Association of American Medical Colleges (2015). Report V: Contemporary Issues in Medicine: Quality of Care.

[CIT0002] National Patient Safety Foundation (2015). Patient Safety Imperative for Health Care Reform. *Position statement issued in October* 2009 *by the Lucian Leape Institute at the National Patient Safety Foundation*.

[CIT0003] Byrne JM, Hall S, Baz S (2012). Quality and safety training in primary care: making an impact. J Grad Med Educ.

[CIT0004] ACGME Common Program Requirements (Section IV.A.5) (2015). https://medicine.umich.edu/sites/default/files/content/downloads/CPRs2013.pdf.

[CIT0005] The Liaison Committee on Medical Education (2015). *Functions and Structure of a Medical School*: 2016-2017.

[CIT0006] Bree KK, Whicker SA, Fromme HB (2014). Residents-as-Teachers Publications: What Can Programs Learn From the Literature When Starting a New or Refining an Established Curriculum?. J Grad Med Educ.

[CIT0007] Qureshi ZU, Gibson KR, Ross MT (2013). Perceived tutor benefits of teaching near peers: insights from two near peer teaching programmes in South East Scotland. Scott Med J.

[CIT0008] Butani L, Paterniti DA, Tancredi DJ (2013). Attributes of residents as teachers and role models-a mixed methods study of stakeholders. Med Teach.

[CIT0009] Institute for Healthcare Improvement (2015). Open School.

[CIT0010] E*Value http://www.webcitation.org/6gZg6VF3X.

[CIT0011] Weber MD, Karman TA. (1991). Student Group Approach to Teaching using the Tuckman Model of Group Development. Am J Physiol.

[CIT0012] IBM Corporation. Released (2012). IBM Statistics for Windows, Version 21.0.

